# Identification of a novel immune-related long noncoding RNA signature to predict the prognosis of bladder cancer

**DOI:** 10.1038/s41598-022-07286-1

**Published:** 2022-03-02

**Authors:** Wenjing Ren, Siyu Zuo, Liang Yang, Renyuan Tu, Ping Wang, Xiling Zhang

**Affiliations:** 1 Department of Urology, The 4th Affiliated Hospital of China Medical University, Shenyang, 110032, China; 2 Liaoning Provincial Key Laboratory of Basic Research for Bladder Diseases, Shenyang, 110000 China

**Keywords:** Cancer, Computational biology and bioinformatics, Urology

## Abstract

Tumour immune regulation has attracted widespread attention, and long noncoding RNAs (lncRNAs) play an important role in this process. Therefore, we evaluated patient prognosis by exploring the relationship between bladder cancer (BLCA) and immune-related lncRNAs (IRlncRNAs). Transcriptome data and immune-related genes were analysed for coexpression, and then, the IRlncRNAs were analysed to determine the differentially expressed IRlncRNAs (DEIRlncRNAs) between normal and tumour samples in The Cancer Genome Atlas. The screened lncRNAs were pairwise paired and combined with clinical data, and finally, a signature was constructed by Lasso regression and Cox regression in 13 pairs of DEIRlncRNAs. According to the Akaike information criterion (AIC) values of the 1-year receiver operating characteristic curve, BLCA patients were stratified into high- or low-risk groups. The high-risk group had a worse prognosis. A comprehensive analysis showed that differences in risk scores were associated with the immune status of BLCA-infiltrated patients. The identified signature was correlated with the expression of immune checkpoint inhibitor-related molecules and sensitivity to chemotherapeutic drugs. We also identified three BLCA clusters with different immune statuses and prognoses that are also associated with immunotherapy response and drug sensitivity. In conclusion, we constructed a powerful predictive signature with high accuracy and validated its prognostic value.

## Introduction

Bladder cancer (BLCA) is the eleventh most frequently diagnosed cancer worldwide. In 2020, U.S. statistics showed that the projected incidence of BLCA was 7%, which makes it the fourth most common cancer in men^1^. In China, the incidence of BLCA has also increased over the past four decades^2^. The main pathological type of BLCA is transitional cell carcinoma. Studies have shown that approximately 25% of patients with BLCA present muscle-invasive BLCA (MIBC), while the remaining 75% have nonmuscle-invasive BLCA (NMIBC)^3^. The recurrence rate of BLCA is high. NMIBC is more likely to recur and develop into MIBC^4^, and MIBC recurs in approximately 50% of cases after radical cystectomy^5^. Currently, the commonly used treatment for NMIBC includes transurethral resection followed by instillation of a drug, such as mitomycin, BCG, and gemcitabine. Studies have shown that such chemotherapy and immunotherapy agents injected into the bladder within 6 h after urethral resection can significantly improve patient prognosis^6^. Common treatment strategies for MIBC include radical cystectomy, neoadjuvant therapy, the use of immune checkpoint inhibitors (ICIs), perioperative radiotherapy, and chemotherapy^1^. Although the prognosis of patients with BLCA has improved with these treatments, recent studies have shown that the use of ICIs can further improve patient outcomes and disease-free survival^7,8^.

Long noncoding RNAs (lncRNAs), which have no protein-coding potential, are RNAs with a transcript length of no less than 200 nt. LncRNAs play important regulatory roles in tissue physiology and various disease processes^9^. The prognosis of many types of cancers is closely related to the status of tumour immune cell infiltration^10–13^. Thus, the role of immune-related lncRNAs (IRlncRNAs) in cancer has also attracted extensive attention. Recent studies have demonstrated that IRlncRNAs can serve as specific biomarkers and can play an important role in predicting prognosis and drug sensitivity in cancer patients^14–17^.

In our study, the expression of IRlncRNAs in BLCA patients was determined. Then, differentially expressed IRlncRNAs (DEIRlncRNAs) were determined by pairing and iterative screening, and finally, an IRlncRNA signature was constructed. This signature eliminates the problem of heterogeneity of different biological samples and different batches in systematic measurement and does not require a specific expression level of each lncRNA. The algorithm of this model is novel in BLCA. Our results demonstrate that this model can be used as a reliable prognostic predictor in patients with BLCA and that patients with different immune statuses can be separated into clusters to evaluate the relationship among tumour immune infiltration, immunotherapeutic responsiveness and the sensitivity of BLCA patients to chemotherapeutic drugs.

## Results

### Data retrieval and extraction

**Figure 1:**
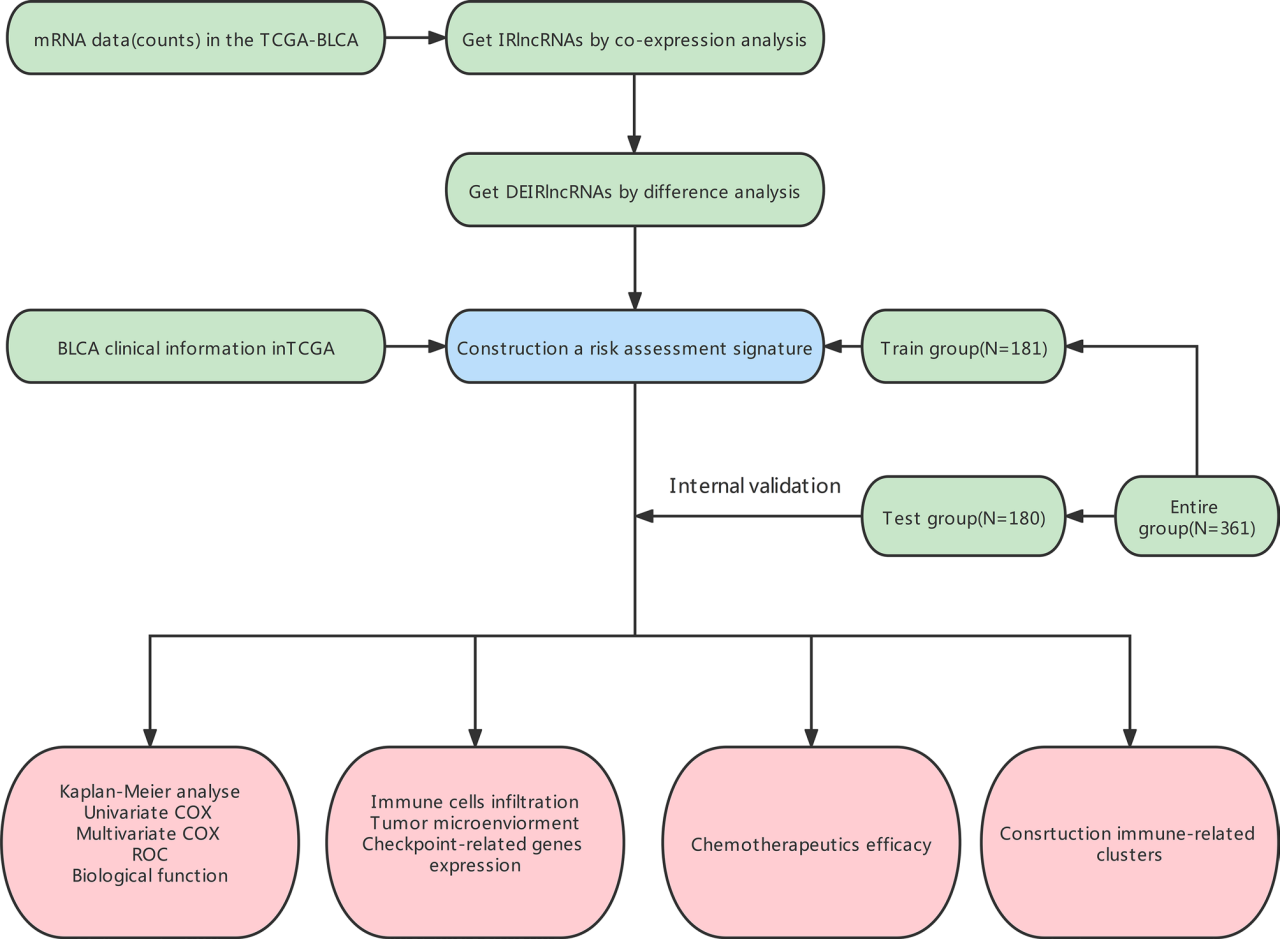
Study flowchart showing the process used for constructing the signature.

**Figure 2:**
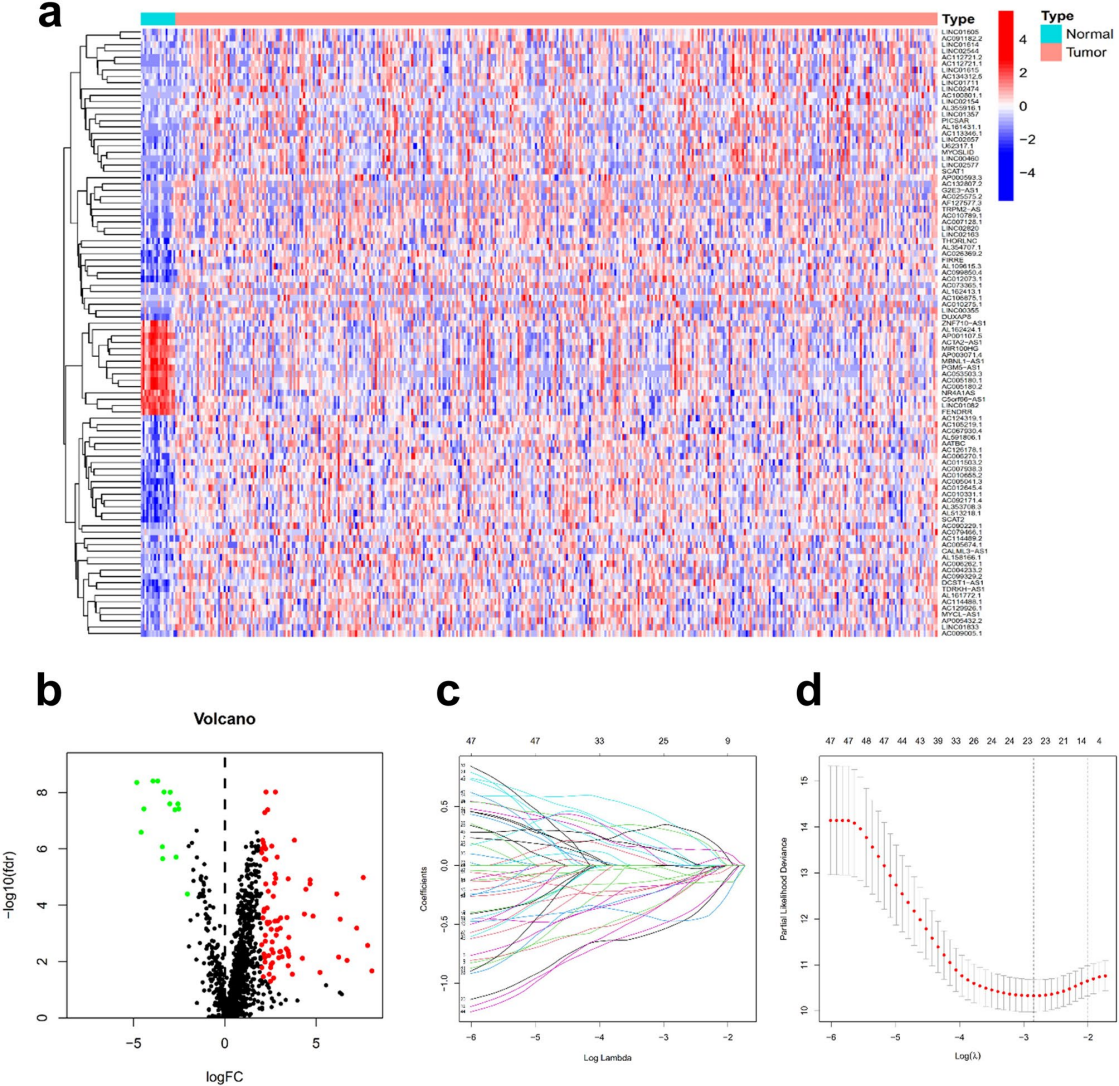
Establishment of an immune-related lncRNA signature. Differentially expressed immune-related lncRNAs (DEIRlncRNAs) were screened using a heatmap (**a**) and volcano plot (**b**). Overall survival (OS) was verified using the least absolute shrinkage and selection operator (Lasso) regression model (**c**) and to elucidate the Lasso coefficient spectrum of prognostic lncRNAs (**d**).

Figure 1 shows the BLCA transcriptome data, which included 18 normal samples and 394 tumour samples downloaded from the TCGA database. LncRNAs were identified following gene annotation and coexpression analysis with immune genes downloaded in Immport to extract IRlncRNAs (Supplementary Table S1). Differential expression analysis was performed on the screened IRlncRNAs, and 96 IRlncRNAs with differential expression were extracted (Fig. 2a). Fifteen IRlncRNAs were downregulated in tumour samples, while 81 were upregulated (Fig. 2b).

### Construction of the immune-related lncRNA signature

Through iterative cycling and 0- or -1 matrix screening, 3890 pairs of DEIRlncRNAs were identified. These 3890 DEIRlncRNA pairs were then correlated with clinical data downloaded from the TCGA database to obtain DEIRlncRNA pairs associated with BLCA prognosis. Using univariate tests and modified Lasso regression analysis (Fig. 2c,d) in the training set (N = 181), 53 pairs of DEIRlncRNAs were obtained (Supplementary Table S2), 13 of which were finally incorporated into the Cox proportional risk model (Supplementary Table S3) to construct the risk model, as described in the “Materials and methods”.

### Validation of the risk prediction model and its value in clinical application

**Figure 3:**
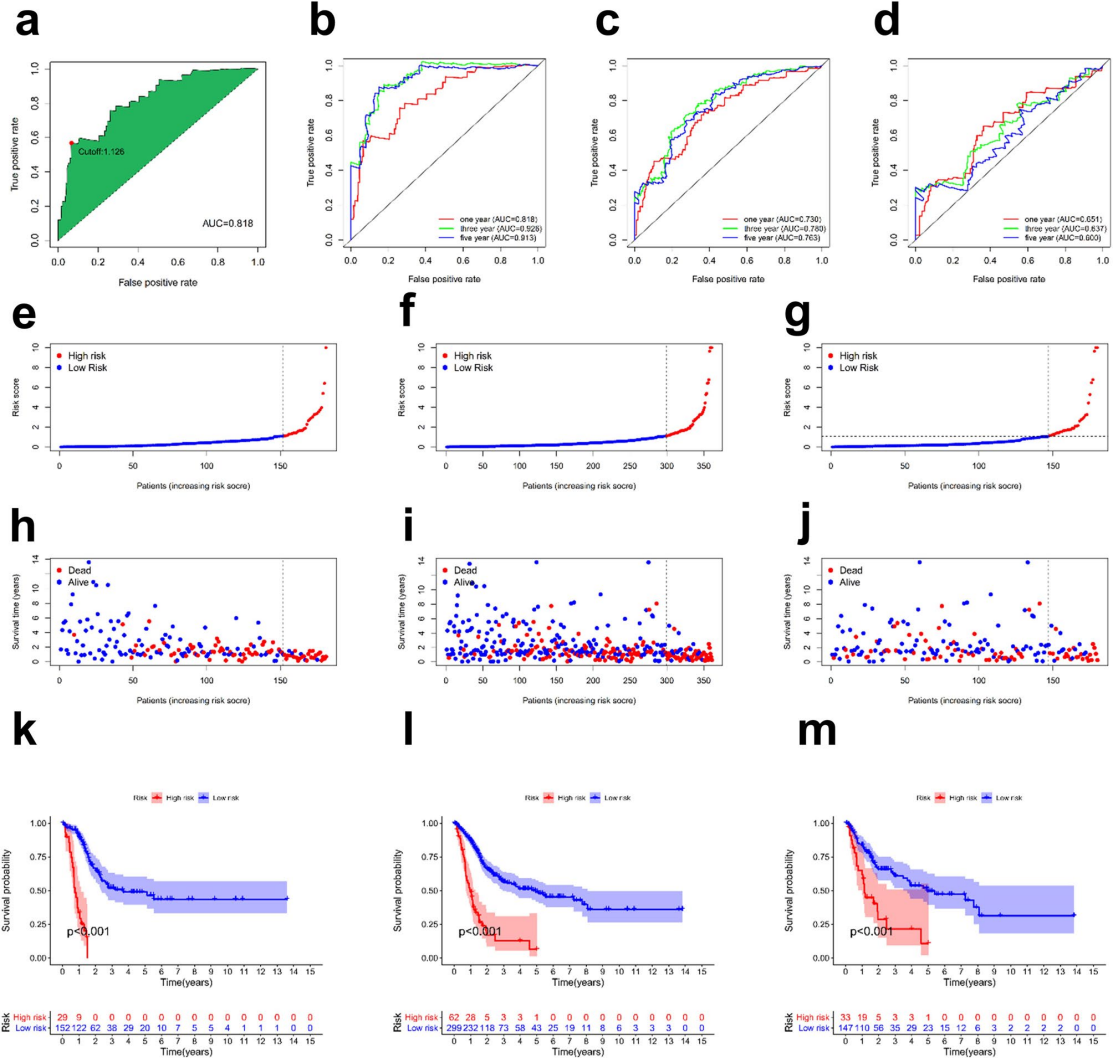
Construction of an immune-related signature to predict the prognosis of BLCA. Receiver operating characteristic (ROC) curve analysis was used to predict overall survival (OS), including the 1-, 3-, and 5-year OS of BLCA patients in the training set (**a**,**b**), entire set (**c**) and test set (**d**). Patients were divided into either the high-risk or low-risk group according to the cut-off point obtained by the Akaike information criterion in the training set (**e**), entire set (**f**) and test set (**g**). Visualization of risk scores and clinical outcomes for each patient in the training set (**h**), entire set (**i**) and test set (**j**). Kaplan–Meier analysis based on risk scores of each BLCA patient was conducted to observe the OS of patients with different risk scores in the training set (**k**), entire set (**l**) and test set (**m**).

Regarding the predictive performance of this model, we constructed ROC curves for the training set and predicted the 1-year overall survival (OS) to be 0.818 (Fig. 3a). In addition, we plotted the ROC curves for the 3- and 5-year OS for the training set (Fig. 3b), and all were above 0.8. Subsequently, we plotted the ROC curves of the entire set and the test set for the 1-, 3- and 5-year survival (Fig. 3c,d). We used the AIC to identify the maximum inflection point as the cut-off value on the one-year ROC curve (Fig. 3a), and a risk score for each patient in all sets was then calculated and compared with a cut-off value; in this manner, each patient was categorized into either the high- or low-risk groups (Fig. 3e–g).

We compared the differences in clinical outcomes between the high- and low-risk groups for all sets (Fig. 3h–j), and over the entire follow-up period, we observed an increase in the mortality rate as the risk score increased. As shown in Fig. 3k–m, Kaplan–Meier analysis indicated that the high-risk group had a shorter OS and that the difference between the survival curves of the two groups with different risk scores was significant.

**Figure 4:**
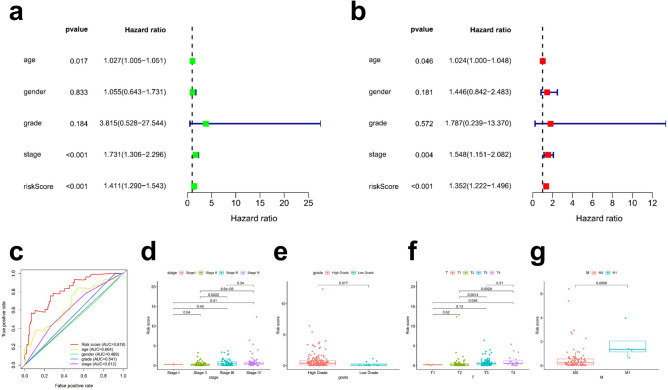
Application of the risk assessment model for clinical evaluation. In the training set, risk assessment demonstrates that the risk model was a risk factor for BLCA by univariate Cox analysis (**a**) and an independent prognostic factor by multivariate regression (**b**). The ROC curves of this model compared with those of other clinical traits (**c**). Clinical stage (**d**), Tumour grade (**e**), T stage (**f**), and M stage (**g**) were significantly associated with the risk score.

To confirm whether this prognostic model can serve as a prognostic risk factor, we tested the association of the model with patient outcomes using Cox regression in the training set (Fig. 4a) compared with other clinical variables, such as age, sex, tumour grade and stage, as covariates. Multivariate Cox analysis confirmed that our risk model was independent of other independent prognostic factors in patients, including clinical characteristics (Fig. 4b). We also verified this finding in the entire set and test set and obtained consistent results (Supplementary Fig. S1).

We compared the ROC curves of this model with those of other clinical traits, as shown in Fig. 4c, which demonstrated that this model is more accurate than other clinical traits in predicting patient prognosis.

To test the relationship between risk and clinicopathological characteristics in patients with BLCA, a chi-square analysis was used. We determined that clinical stage (Fig. 4d), tumour grade (Fig. 4e), T stage (Fig. 4f), and M stage (Fig. 4g) were significantly correlated with the risk score. In conclusion, our risk assessment model can serve as an independent prognostic factor for bladder cancer.

### Correlation analysis of tumour-infiltrating immune cells and immunosuppressive molecules using a risk assessment model

**Figure 5:**
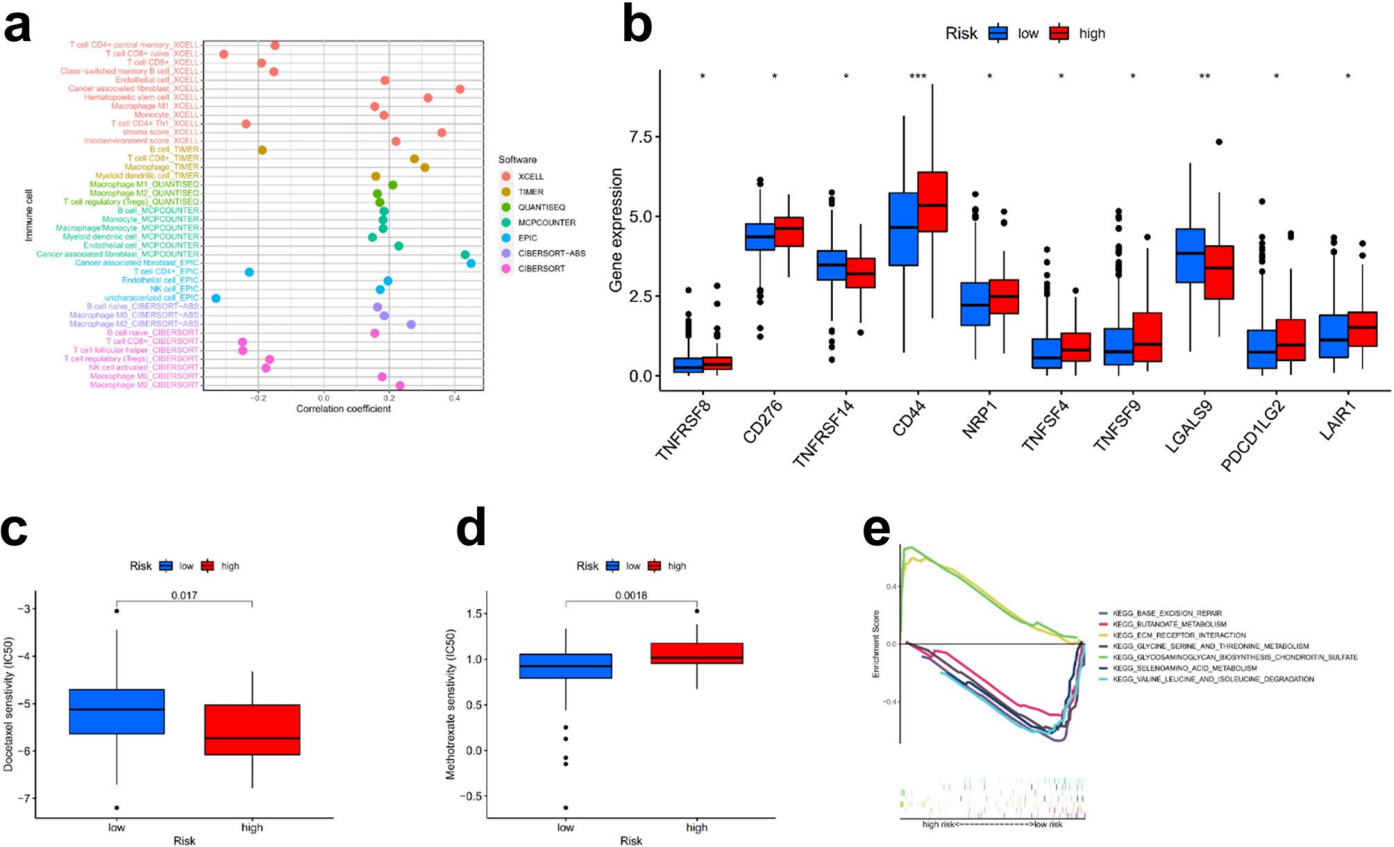
Risk assessment model evaluation of tumour-infiltrating cells, immunosuppressive molecules and biological function. Spearman correlation analysis was used in the training set to demonstrate differences in the levels of tumour-infiltrating cells across different risk groups, and the results are shown in a lollipop diagram (**a**). Differentially expressed immune checkpoints in the high- and low-risk groups (**b**). Drug sensitivity to docetaxel (**c**) and methotrexate (**d**) in the high- and low-risk groups. GSEA of the training set (**e**).

Since the differential lncRNAs identified in our study were immune-related, we speculated that this risk assessment model might be related to the tumour immune microenvironment. Using the Wilcoxon signed-rank test, the high-risk group was found to have certain tumour-infiltrating immune cells, such as cancer-associated fibroblasts (CAFs)^18,19^, haematopoietic stem cells and macrophages. However, the model was negatively correlated with CD4 + Th1 T cells, Tregs and naive CD8 + T cells (Supplementary Fig. S2). The results of the Spearman correlation analysis of these platforms mentioned in the “Materials and methods” are shown in Fig. 5a, and the details of each immune cell type are shown in Supplementary Table S4. In parallel, we also performed the same analysis for the entire group and the test group and obtained similar results (Supplementary Fig. S3, S4 and Supplementary Table S5, S6). As reflected in the figure, the high-risk group had more tumour infiltration of immune cells.

Tumour immunotherapy has developed rapidly in recent years. Several clinical trials to determine the response of urothelial carcinoma to immune checkpoint inhibitors are ongoing and have yielded promising results^20,21^; moreover, ICIs targeting PD-1 and CTLA-4 have been shown to significantly improve the prognosis of locally advanced and advanced BLCA and are well tolerated^22–24^. For this reason, we investigated whether this signature was associated with certain biomarkers associated with ICIs. As illustrated in Fig. 5b, the expression of some immune checkpoint inhibitors was different in the high- and low-risk groups, and thus, we may be able to find suitable immune checkpoint inhibitors for different groups of bladder cancer patients.

### Correlation analysis between the risk assessment model and chemotherapeutic drug sensitivity and biological function

In addition to tumour ICI therapy, the correlation between the risk assessment model and sensitivity to conventional chemotherapy was also evaluated. Patient risk scores were associated with sensitivity to certain conventional chemotherapeutic agents. The IC50 values of 26 chemotherapy drugs were different between the high- and low-risk groups of the training set (Supplementary Fig. S5). For example, the high-risk group was more sensitive to the chemotherapy drug docetaxel (Fig. 5c) and less sensitive to methotrexate (Fig. 5d). In parallel, we also performed the same analysis for the entire group and the test group and obtained similar results (Supplementary Fig. S6, S7). Thus, the risk model may be used as a predictor of chemotherapy sensitivity. In addition, we explored the biological function of this model through GSEA in the training set (Supplementary Fig. S8) and found that the pathways enriched in the high-risk group were correlated with ECM receptor interactions and glycosaminoglycan biosynthesis and chondroitin sulfate. The low-risk group was correlated with base excision repair and selenoamino acid metabolism (Fig. 5e).

### Construction of immune subtypes in bladder cancer

**Figure 6:**
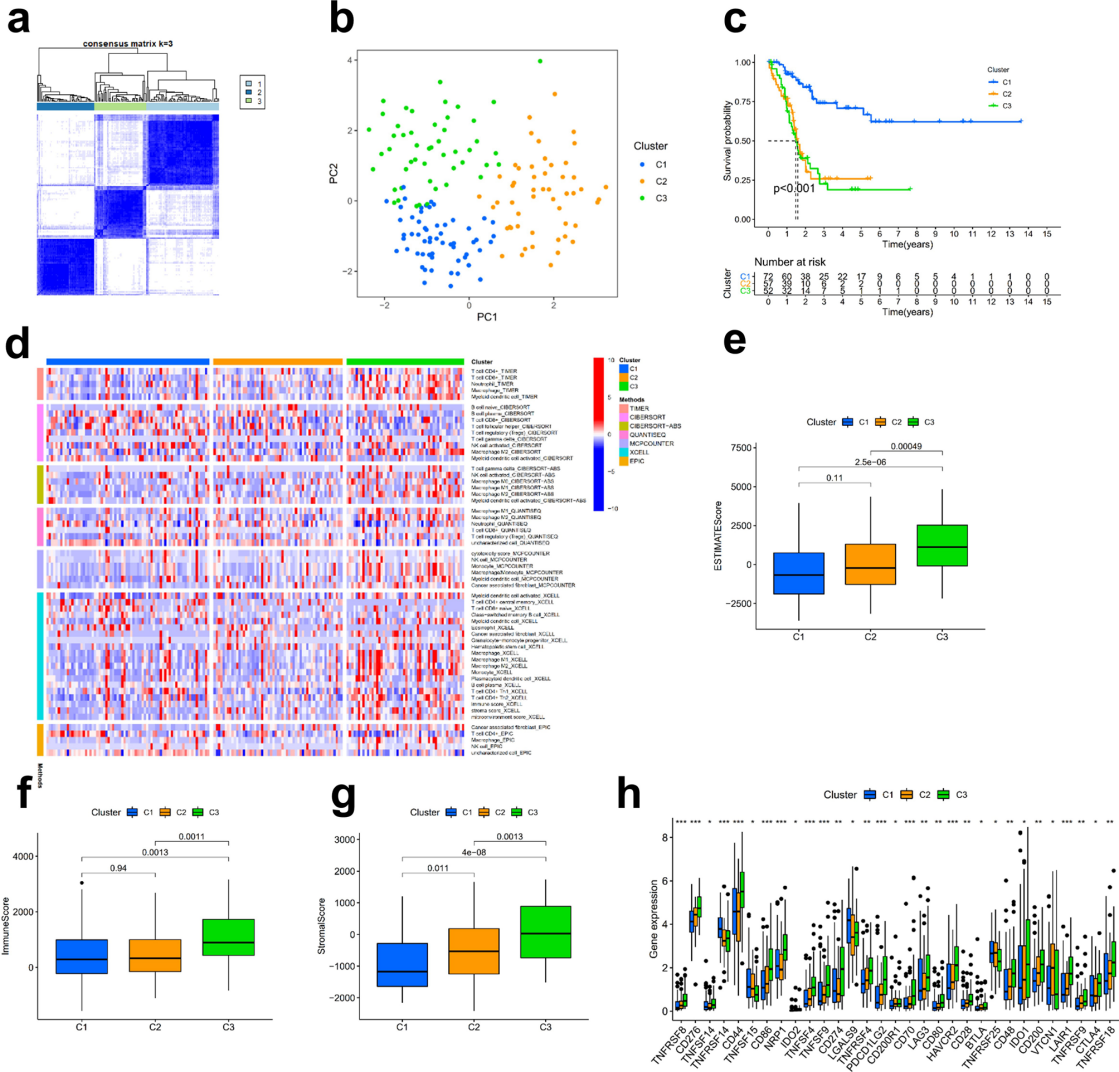
Construction, validation and immunocorrelation analysis of BLCA clusters. Different clusters of patients with bladder cancer (**a**). PCA was performed on the clusters (**b**). Kaplan–Meier analysis based on risk scores of each BLCA patient was conducted to observe the OS of patients in different clusters (**c**). Immune cell infiltration among different clusters (**d**). The ESTIMATEScore (**e**), ImmuneScore (**f**) and StromalScore (**g**) of each cluster. Differentially expressed immune checkpoint proteins in the three clusters (**h**).

These results show that tumours can be divided into different molecular subtypes according to immune cell infiltration and that this classification is closely related to immunotherapy response and clinical outcomes^25–27^. Thirteen pairs of DEIRlncRNAs are the basis for our ability to reclassify bladder cancer patients into three clusters. This process was completed using *R* package ConsensusClusterPlus consensus cluster analysis of the training cohort (Fig. 6a and supplementary S9). To ensure that different clusters could be well defined, we used principal component analyses (PCA) for verification, and the results were as expected (Fig. 6b). Kaplan–Meier analysis revealed a significant difference in prognosis between patients in Cluster 1 and those in the other two clusters, and patients in Cluster 1 had the best prognosis (Fig. 6c). To verify the differences in immune cell infiltration and the TME among different clusters, we first plotted the results of immune cell infiltration of each cluster obtained on different platforms, and Cluster 3 was found to have the most obvious immune cell infiltration (Fig. 6d). Second, according to the results of the ESTIMATE algorithm, the ESTIMATEScore, ImmuneScore and StromalScore of Cluster 1 were lower than those of the other two clusters (Fig. 6e–g). Subsequently, we mapped immune checkpoint proteins, such as such as CTLA4, LAG3, and HAVCR2, that were expressed differently in different clusters and found that the majority were most highly expressed in Cluster 3 (Fig. 6h). For this purpose, we may be guided by these results when selecting suitable immune checkpoint inhibitors for different patient clusters to improve response to immunotherapy. With *P* < 0.05 as the filtering condition, 89 chemotherapy drugs with IC50 differences between clusters were obtained (Supplementary S5). Generally, the classification of BLCA patients into different clusters according to the 13 pairs of DEIRlncRNAs can help us predict immunotherapy response to a certain extent and can provide guidance for selecting more sensitive drugs for different clusters.

### Comparison of the efficiency of the risk model of paired DEIRlncRNAs and the signature constructed with 11 immune-related lncRNAs

**Figure 7:**
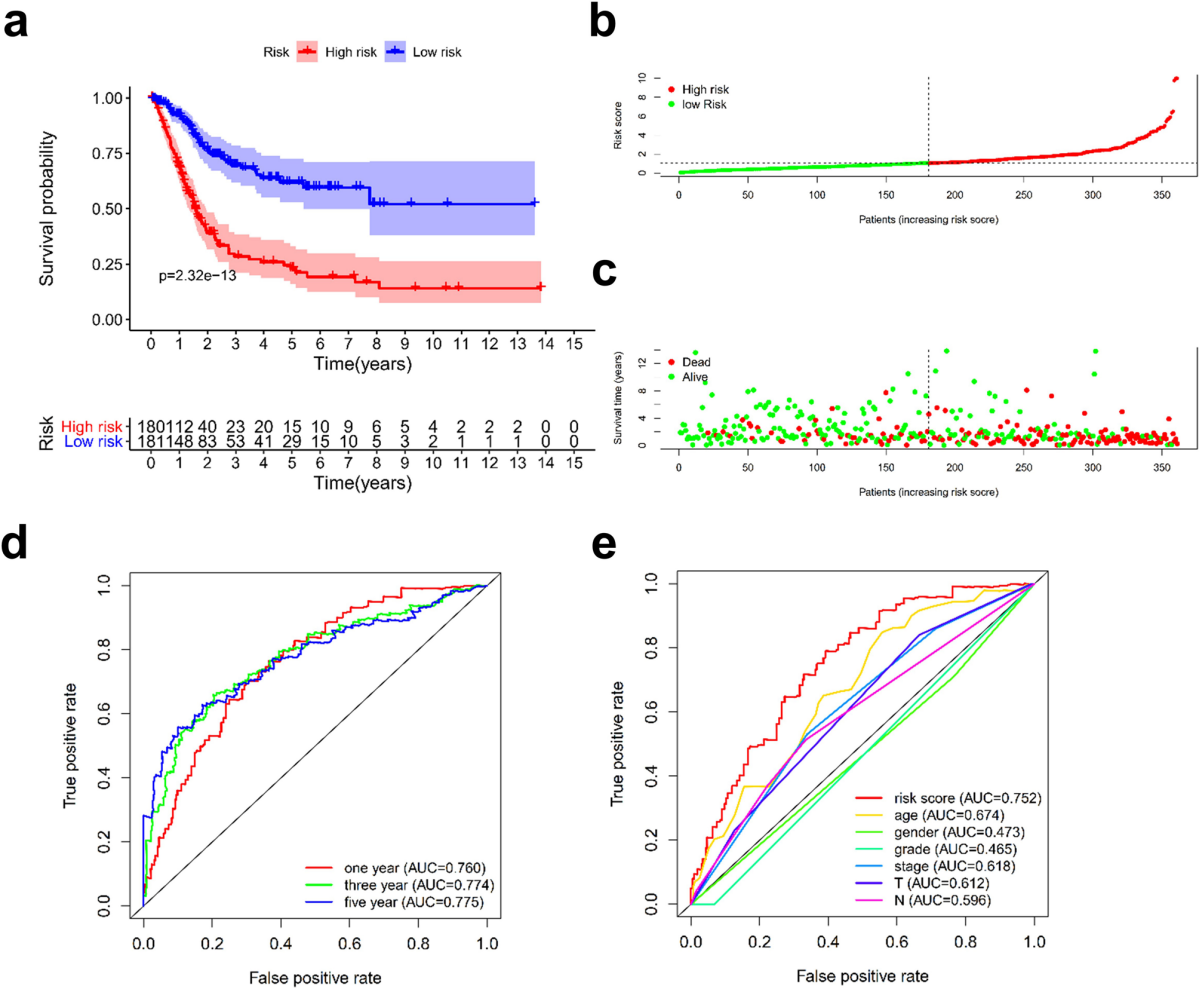
Validation of the effectiveness of the 11 immune-related lncRNA signature. Kaplan–Meier analysis was performed by stratifying BLCA patients according to risk scores to observe the OS of patients (**a**). Risk scores and clinical outcomes for each patient (**b**,**c**). The ROC curves of this model compared with those of other clinical traits (**d**). The AUC values of the ROC, including the 1-, 3-, and 5-year OS of BLCA patients (**e**).

We applied a commonly used method for model construction. First, we screened IRlncRNAs associated with prognosis and then established a prognostic model based on the expression of each lncRNA through multiple Cox regression analysis. In this signature, patients were divided into low-expression groups based on the median risk score to compare whether our prognostic signature was superior to other immunoprognostic models. Based on the above methods, we constructed an 11-IRlncRNA signature (Supplementary Table S7). The differences in clinical outcomes between the high- and low-risk groups of this model and their relationship with OS were visualized, and the 1-, 3-, and 5-year ROC curves were drawn. This model also demonstrated a certain prognostic predictive ability, as the prognoses of the high- and low-risk groups were significantly different (Fig. 7a–c). Compared with more well-known clinical characteristics of patients with BLCA (Fig. 7d), the model demonstrated better prognostic ability despite the 1-, 3-, and 5-year ROC curve values and despite that the respective AUC values (Fig. 7e) were 0.760, 0.774, and 0.775, respectively. Nonetheless, the prediction accuracy of this model was lower than that of our model constructed using the paired IRlncRNA signature.

## Discussion

BLCA is a common malignant tumour of the urinary system. Although the active use of various treatment methods has improved the survival rate of patients with BLCA, the treatment outcomes are still not satisfactory^28^. Therefore, biomarkers that can predict the prognosis and drug sensitivity of BLCA are urgently needed. In recent years, lncRNAs, which play an important role in cell functions, including tumour migration, invasion, growth and development, have also been found to serve as potential biomarkers for predicting tumour prognosis^17,29–31^. Although several lncRNA-based signatures can predict prognosis in patients with BLCA, most signatures are based on the specific expression of a single lncRNA^32–35^. If the expression of a single lncRNA is required, normalization is needed across different samples and batches to eliminate heterogeneity. In this study, paired DEIRlncRNAs were used for the first time to construct a risk assessment model in BLCA. Using this model, we only compared the relative expression of two DEIRlncRNA pairs in the sample since batch correction was not necessary.

Before model construction, we randomly divided patients into a training group and a test group. Using the model, which was constructed based on 13 pairs of DEIRlncRNAs in our training group, patients with BLCA can be divided into different risk groups according to their risk scores. Kaplan–Meier analysis, ROC curve analysis and other methods were used to verify the accuracy of our model. Moreover, this model was superior to other clinical parameters in predicting survival. Cox regression analysis confirmed that this model is an independent prognostic factor. The above results demonstrate that this model can accurately predict patient prognosis. In addition, we predicted the level of tumour-infiltrating immune cells. We found that the risk score was positively associated with infiltration by macrophages, cancer-associated fibroblasts and other cell types. Studies have shown that macrophages play an important regulatory role in promoting malignant tumour progression^36^, while LNMAT1 promotes lymphatic metastasis of BLCA through recruitment of CCl2-dependent macrophages^37^, and BMP4 induces polarization of M2 macrophages and facilitates tumour progression in BLCA^38^. CAFs are closely associated with cancer progression and are present at the highest levels in the stroma. CAFs induce epithelial-mesenchymal transition of BLCA cells through paracrine IL-6 signalling^39^. Appropriate chemotherapy drugs can also be selected according to the patient's risk score. These results were also verified internally in the test set and the entire set.

During tumorigenesis and tumour progression, immune checkpoints are one of the main causes of immune tolerance. However, we found that only some of the immune checkpoint were differentially expressed in the high- and low-risk groups, which may lead to inadequate prediction of immunotherapy response in bladder cancer patients. The effect of immunotherapy may be closely related to the immune microenvironment^40–42^. Some recent studies have shown that tumours can be divided into different immune clusters according to different infiltration states of immune cells, which can be more effective in predicting the TME of tumours and thus better in predicting the response to immunotherapy^25,27,42–44^. Therefore, we divided bladder cancer patients into 3 clusters based on the 13 pairs of DEIRlncRNAs. The ESTIMATEScore, ImmuneScore and StromalScore of the TME were calculated. The results show that this method can clearly distinguish the TME of the three clusters. Cluster 1 had the lowest ESTIMATEScore, ImmuneScore and StromalScore and had the best prognosis and the lowest level of tumour immune cell infiltration. Through the clusters of BLCA, we found more immune checkpoint proteins that were differentially expressed beyond what was shown by the risk assessment model. This enabled us to select the curative effect of immune checkpoint inhibitors in patients with BLCA and to better predict the response of BLCA patients to immunotherapy, as well as to select the corresponding sensitive chemotherapeutic drugs for each cluster.

The signature constructed by paired DEIRlncRNAs showed stronger predictive power than the signature constructed by the more commonly exploited single gene expression approaches, although both approaches showed some predictive power.

However, due to the limited amount of BLCA data in the TCGA database, the signature we constructed has some limitations. For example, as the Gene Expression Omnibus (GEO) and other databases mainly focus on the expression of coding RNA, few relevant lncRNA data are published, which makes it diffcult for us to verify the risk model we established using external datasets. Moreover, we lack information regarding the exact molecular mechanisms of the IRlncRNAs used to construct the model. In future studies, we will collect clinical samples and conduct external validation of our model. Moreover, the investigation of the cellular mechanisms and processes involving screening lncRNAs should be studied using single or paired methods.

Overall, we constructed a signature that may potentially be used as an independent predictive indicator of prognosis in patients with BLCA. The model consists of 13 pairs of DEIRlncRNAs. We verified that this model can assess the status of immune infiltration to a certain extent and was correlated with the efficacy of selected chemotherapeutics. Thus, we hope that this model may help identify patients with BLCA who have a poor prognosis, provide guidance for treatment selection, and ultimately improve the prognosis of patients with BLCA.

## Materials and methods

### Datasets of patients with bladder cancer

The transcriptome, including BLCA RNA-Seq data and related clinical data, was downloaded from The Cancer Genome Atlas (TCGA) database. These data were then merged into a matrix file using the Perl programming language. Clinical data with zero follow-up days were excluded. Human immune-related genes were downloaded from the ImmPort^45,46^ project.

### Differentially expressed immune-related lncRNAs

To identify IRlncRNAs, we first used the *R* package limma^47^ to analyse the coexpression of immune-related genes and lncRNAs with the following screening conditionscorrelation coefficient > 0.4 and *P* value < 0.001. To identify DEIRlncRNAs, the *R* packages limma and pheatmap were used for differential expression analysis of IRlncRNAs among the normal and cancer samples downloaded from TCGA. The threshold was set to |log2-fold change [FC]| > 2 and a false discovery rate (FDR) < 0.05.

### Determination of paired DEIRlncRNAs

A 0 or 1 expression matrix was constructed by pairing DEIRlncRNAs and comparing two DEIRlncRNAs in each pair. When the expression level of the first lncRNA was higher, the value was marked as 1; otherwise, it was marked as 0. The 0 or 1 matrix was then screened for DEIRlncRNA pairs, and those with a 0 or 1 ratio greater than 80% were deleted.

### Establishment of a Risk Assessment Model

Using the *R* package caret, the TCGA-BLCA dataset was allocated randomly to the training set or the test set at a one-to-one ratio^48,49^. In the training set, univariate Cox analysis was performed to identify DEIRlncRNAs associated with prognosis, followed by Lasso regression, 10-fold cross-validation, and finally, stepwise multivariate Cox proportional risk regression analysis and model construction. The *R* packages survival, survminer, and glmnet were used^11^.

### Validation of the risk assessment model

Prognostic value verification was performed using the test set and the entire set. The risk score for each patient was calculated according to the following formula:

$${\text{Risk}}\,{\text{Score}} = \mathop \sum \limits_{i = 1}^{k} \beta iSi$$

The regression coefficient was denoted by $\beta$, and the score of immune-related lncRNA pairs in the sample was denoted by $S$. In the training set, the Akaike information criterion (AIC) value of each point in the 1-year ROC curve was evaluated to determine a cut-off value, and patients in each set were divided into either the high- or low-risk groups according to the calculated cut-off value obtained in the training set. The receiver operating characteristic (ROC) curves of all sets for this model at different time points and clinical traits of patients with BLCA were plotted. The area under the curve (AUC) was used to test the efficacy of this model in predicting the prognosis of patients with BLCA. The clinical outcomes associated with each sample were observed to identify any differences in outcomes between high- and low-risk patients. We used a Kaplan–Meier analysis to understand and visualize differences in OS between the two groups. The R packages survival and survminer were used. We analysed the relationship between the model with the clinicopathological characteristics using the chi-square test to determine the potential clinical value of the model. The R packages limma and ggpubr were used for this analysis.

### Evaluation of tumour-infiltrating immune cells and expression analysis of ICI-related immunosuppressive molecules

Immune cell infiltration estimation data for all cancers in the TCGA were downloaded from TIMER2.0^50^ (http://timer.cistrome.org/). We used currently established algorithms (xcell, timer, quantiseq, mcpcounter, epic, cibersort-abs, and cibersort^51^) to predict the relationship between these immune cell characteristics and risk. The results of Spearman’s correlation analysis (*P* < 0.05) and the relationships identified are displayed in a lollipop diagram^17^.

The Wilcoxon signed-rank test was used to analyse differences in the expression of infiltrating immune cells between the high- and low-risk groups, and the results are presented in a box diagram. The *R* packages limma, scales, ggplot2, reshape2, tidyverse, ggpubr and ggtext were used. The *R* packages ggpubr and reshape were used to illustrate the relationship between this model and the expression levels of ICI-related genes.

### Evaluation of the significance of this model in clinical treatment

First, to understand the potential significance of this model in the clinical treatment of BLCA, we used the Wilcoxon signed-rank test. First, the half inhibitory concentration (IC50) of each BLCA patient was determined using the package “pRRophetic^52^”, which is a drug response prediction algorithm based on the Genomics of Drug Sensitivity in Cancer (GDSC). Second, we compared the IC50 values of these drugs for both the high- and low-risk groups. The samples used were derived from the BLCA dataset extracted from the TCGA database. The *R* packages used were pRRophetic^52,53^ limma, ggpubr, and ggplot2.

### Biological function analysis of this model

Gene set enrichment analysis (GSEA) was the method used for Kyoto Encyclopedia of Genes and Genomes (KEGG) pathway analysis in the entire set. Gene sets for which P < 0.05 were screened.

### Consensus clustering based on 13 pairs of DEIRlncRNAs

To further explore the response of bladder cancer patients to immunotherapy, we performed a cluster analysis to classify bladder cancer into different molecular subtypes. This process was performed using the *R* package ConsensusClusterPlus^54^ and was based on 13 pairs of DEIRlncRNAs that were used to construct the risk assessment model mentioned above. Differences in the ESTIMATE score, immune score, and stromal score among different subtypes of bladder cancer were estimated by running the *R* package ESTIMATE^41^.

### Construction of the immune-related lncRNA signature

The identification of IRlncRNAs used in the signature is described above. Univariate Cox regression was performed to screen for IRlncRNAs associated with prognosis by filtering those for which *P* < 0.001. Stepwise regression multivariate Cox analysis was then performed to establish the risk model as follows:

$$\begin{aligned} {\text{RiskScore}} & \, = \, \left( {{\text{expressionlncRNA1 }} \times {\text{ coefficientlncRNA1}}} \right) \\ & \quad + ({\text{expression lncRNA2}} \times {\text{ coefficientlncRNA2}}) + \ldots \\ & \quad + \left( {{\text{expressionlncRNAn }} \times {\text{ coefficientlncRNAn}}} \right) \\ \end{aligned}$$

The median risk score was used to divide patients into high- and low-risk groups. Kaplan–Meier curves were plotted based on the risk scores of patients with BLCA. The risk scores of patients with BLCA were visualized to observe differences in clinical outcomes. Univariate Cox analysis and multivariate Cox regression were used to determine whether the IRlncRNA signature could serve as an independent risk factor. The ROC curves of the model at 1, 3, and 5 years were plotted, as were the ROC curves of the model against clinicopathological characteristics. The *R* packages used were survival, survminer, pheatmap, and survivalroc.

### Ethics committee approval and patient consent

All data used in this article are from the public TCGA database and were obtained in compliance with the ethical standards of the "Gene Expression Synthesis" and The Cancer Genome Atlas Human Subject Protection and Data Access Policy" adopted by the National Cancer Institute and the National Human Genome Research Institute.

## Supplementary Information

Supplementary Information 1.

Supplementary Table S1.

## Data Availability

The original contributions presented in the study are publicly available. These data can be found at [https://portal.gdc.cancer.gov/]. The immune-related gene list can be found at [http://www.immport.org]. The data of Immune cell infiltration estimation can be found at [http://timer.cistrome.org/].

## Abbreviations

BLCA: Bladder cancer
LncRNAs: Long noncoding RNAs
IRlncRNAs: Immune-related lncRNAs
TCGA: The Cancer Genome Atlas
DEIRlncRNA: Differentially expressed immune-related long noncoding RNAs
AIC: Akaike information criterion
AUC: Area under the curve
ROC: Receiver operating characteristic
HR: Hazard ratio
GSEA: Gene Set Enrichment Analysis
KEGG: Kyoto Encyclopedia of Genes and Genomes
