# Supplementary material for: Identification of a novel immune-related long noncoding RNA signature to predict the prognosis of bladder cancer
Source: Sci Rep. 2022 Mar 2;12:3444. doi: 10.1038/s41598-022-07286-1 (PMC8891323; doi:10.1038/s41598-022-07286-1)
Supplement: Supplementary file 1 — Supplementary Information 1. [file 41598_2022_7286_MOESM1_ESM.pdf]

Identification of a Novel Immune-Related Long Noncoding RNA Signature to Predict Prognosis of Bladder Cancer

Wenjing Ren[1,2], Siyu Zuo[1,2], Liang Yang[1,2], Renyuan Tu[1,2], Ping Wang[1,2], Xiling Zhang[1,2*]

[1]The 4th affiliated hospital of China Medical University, Department of Urology, Shenyang, 110000, China

[2]Liaoning Provincial Key Laboratory of Basic Research for Bladder Diseases, Shenyang, 110000, China

[*]corresponding. xilingzhang@cmu.edu.cn

Supplementary Figures and Tables

Supplementary Figures

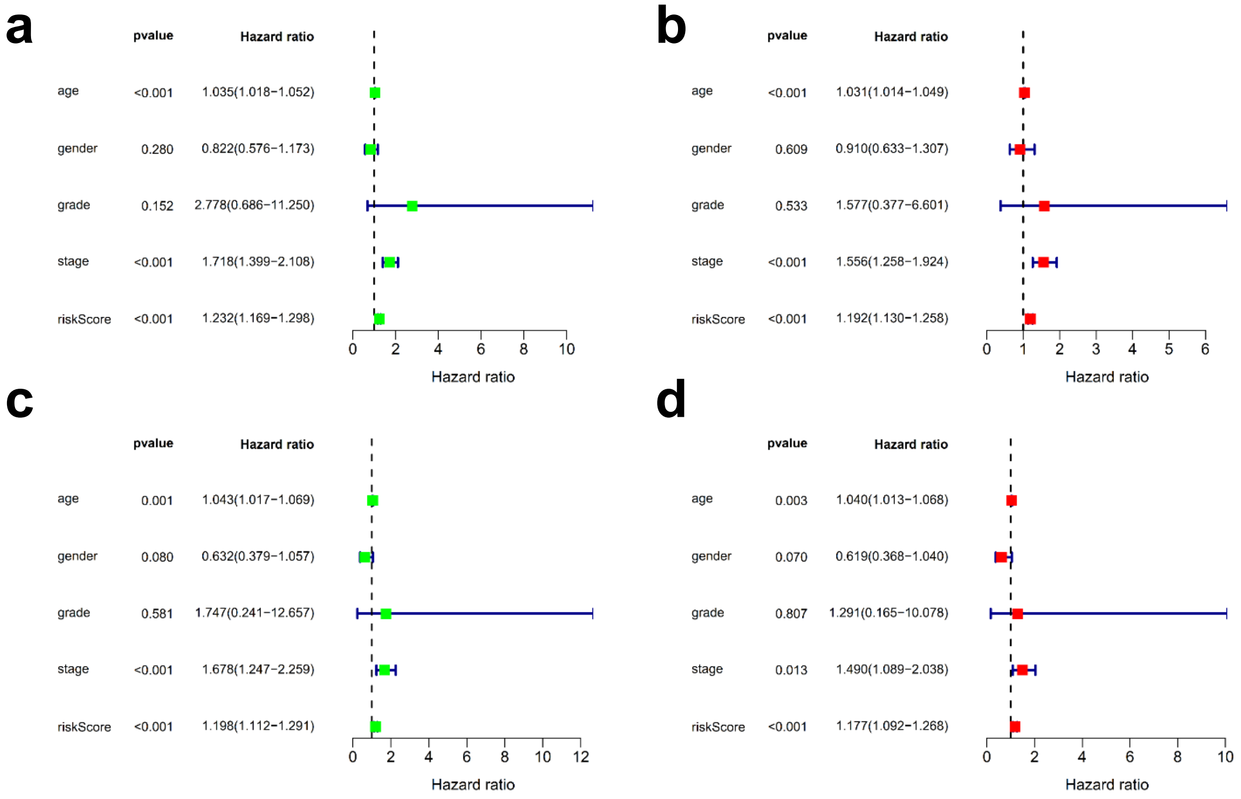

Supplementary Figure 1: Application of the risk assessment model for clinical evaluation in the entire set and the test set

Univariate Cox analysis (a) and multivariate regression (b) for the entire set**,** univariate Cox analysis (c) and multivariate regression (d) for the test set.

Supplementary Figures

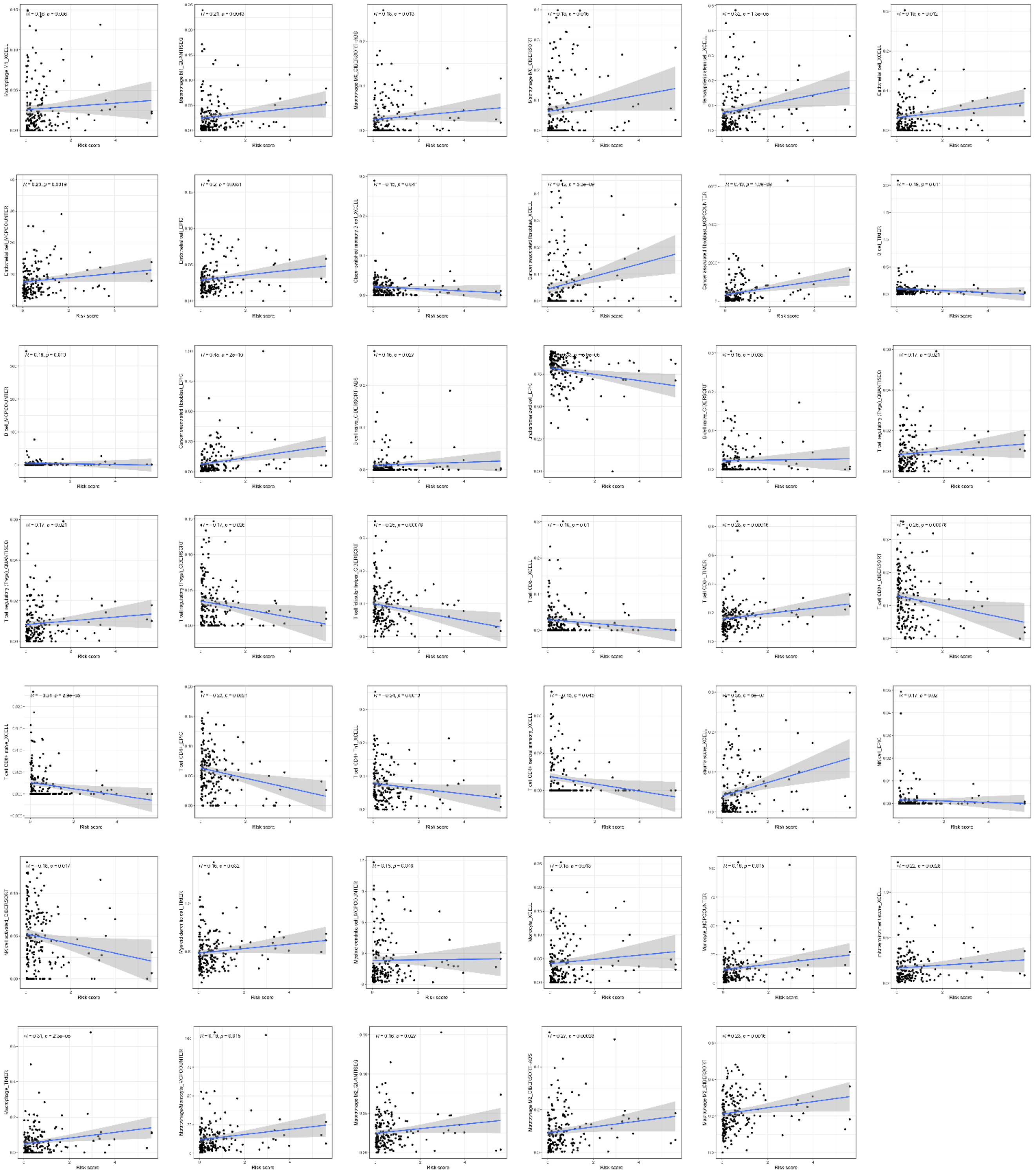

Supplementary Figure 2: Correlation analysis of tumour-infiltrating immune cells in the training set

Supplementary Figures

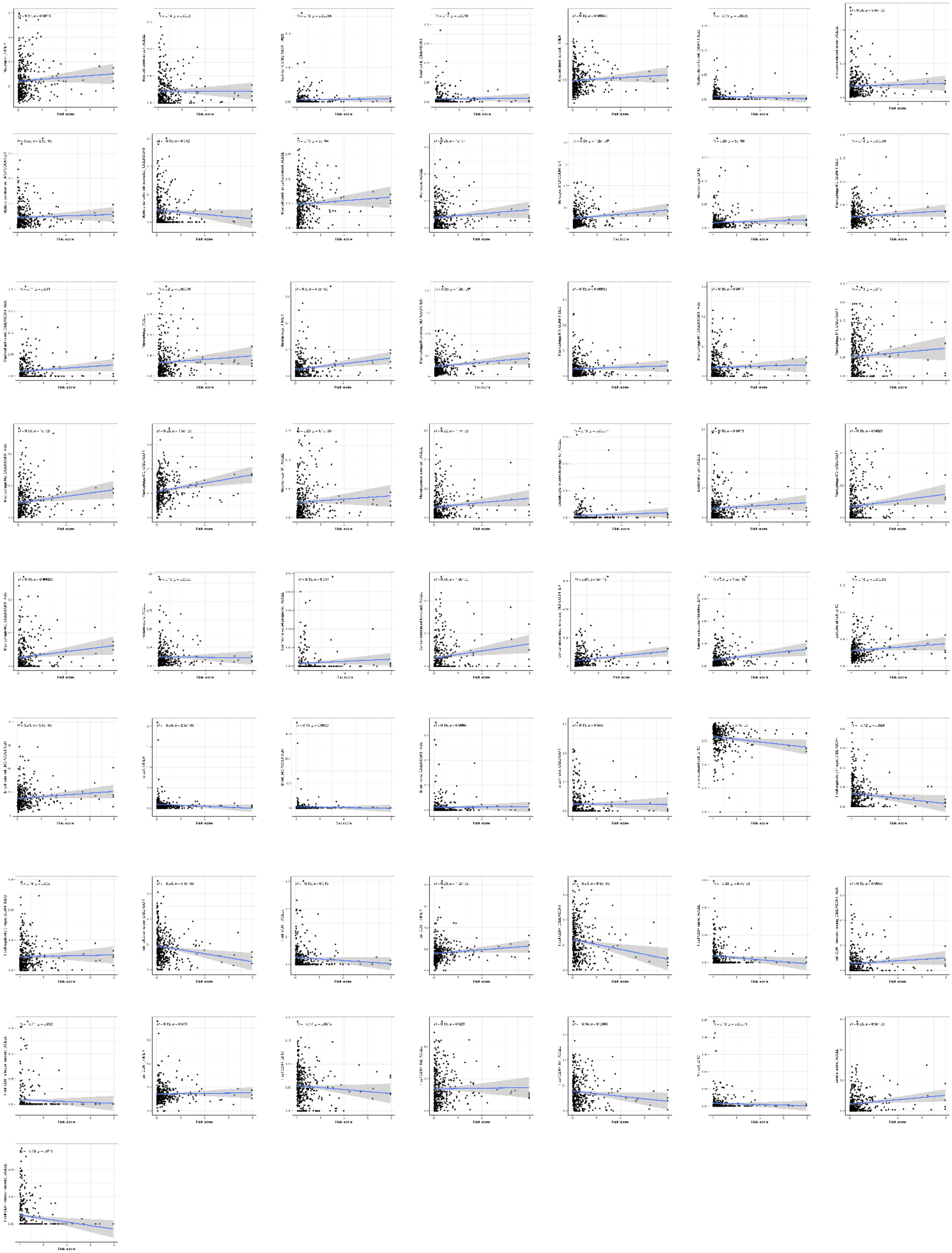

Supplementary Figure 3: Correlation analysis of tumour-infiltrating immune cells in the entire set

Supplementary Figures

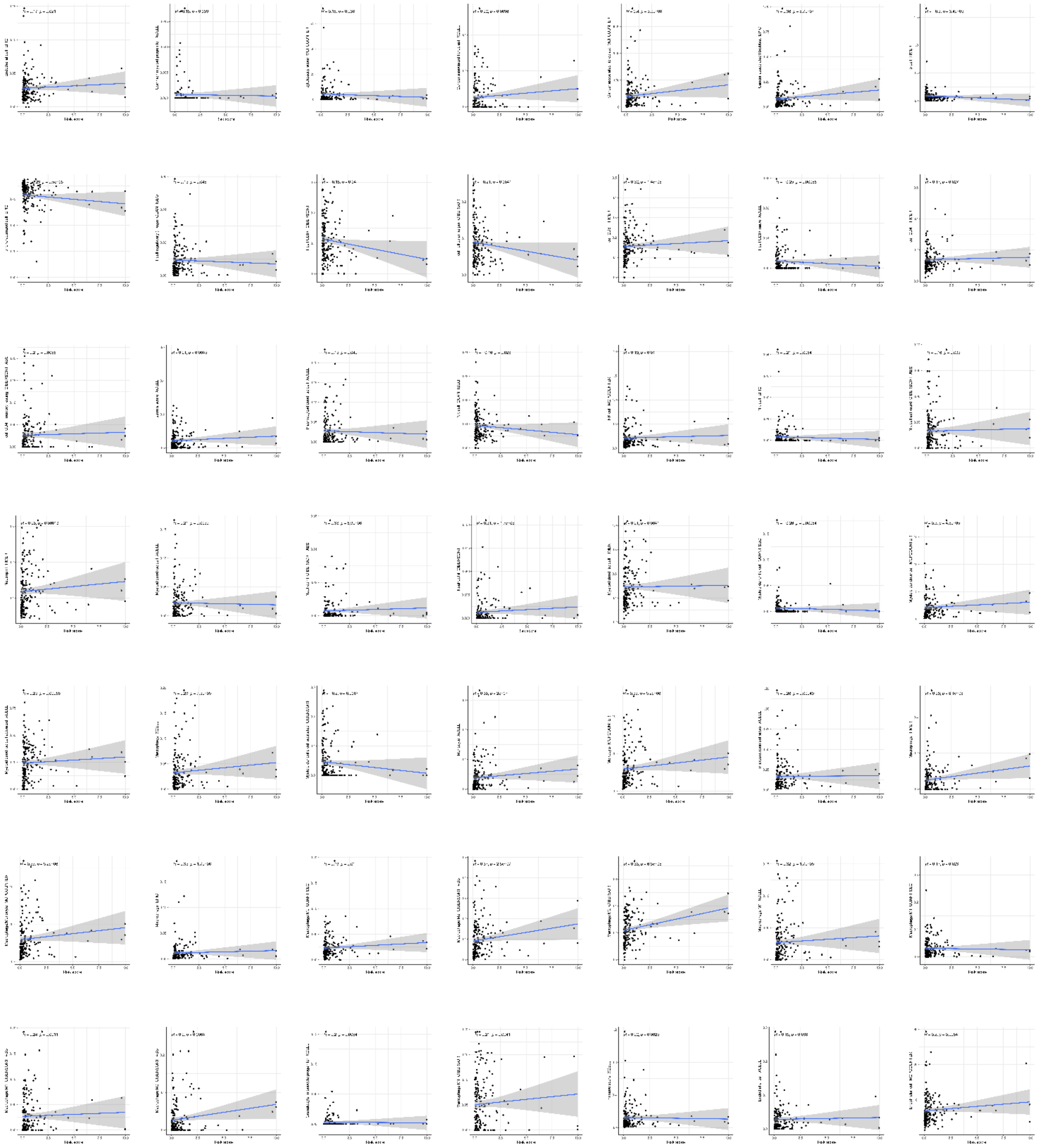

Supplementary Figure 4: Correlation analysis of tumour-infiltrating immune cells in the test set

Supplementary Figures

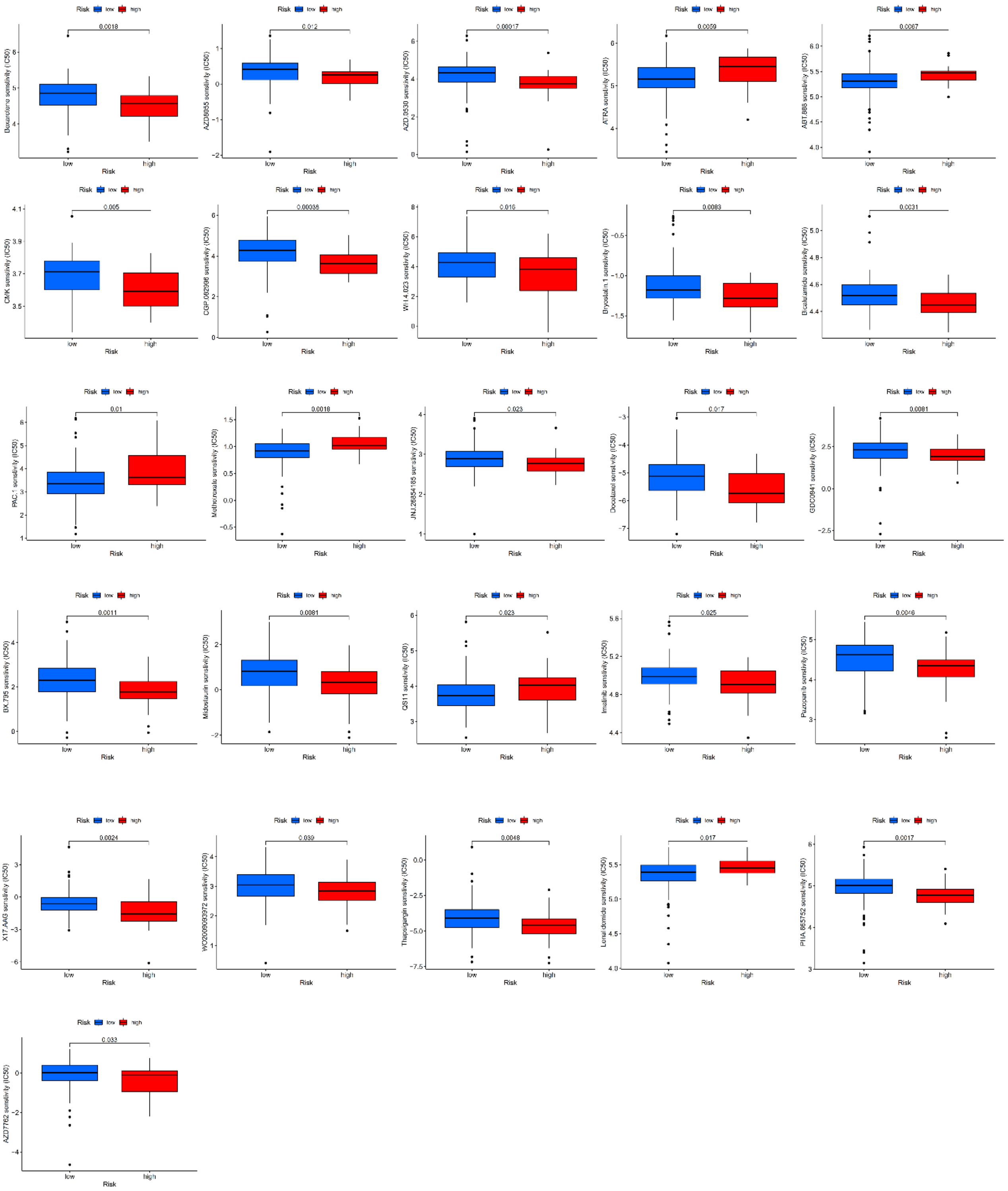

Supplementary Figure 5: The IC50 values of 26 chemotherapy drugs were different between the high- and low-risk groups of the training set

Supplementary Figures

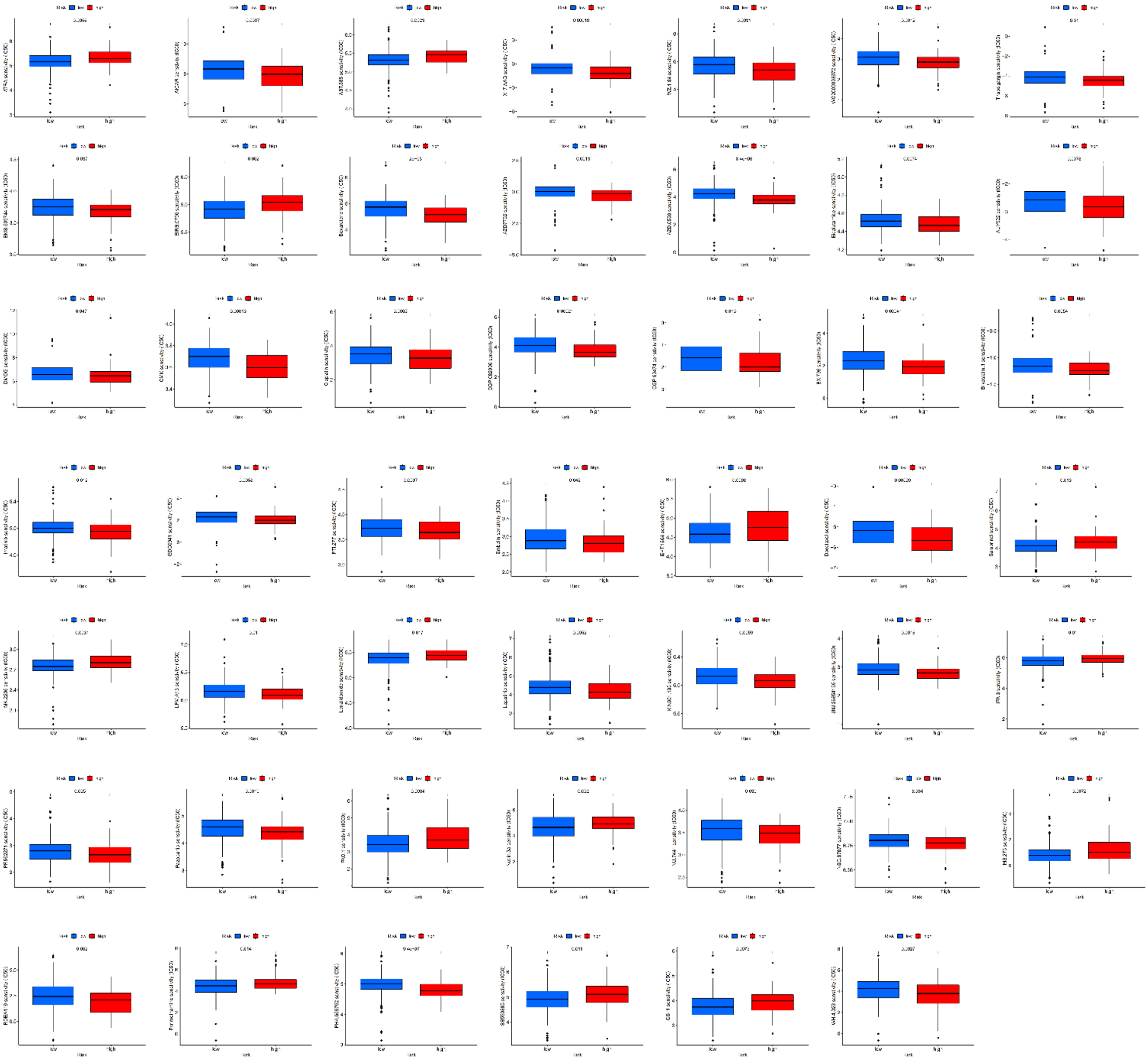

Supplementary Figure 6: The IC50 values of 48 chemotherapy drugs were different between the high- and low-risk groups of the entire set

Supplementary Figures

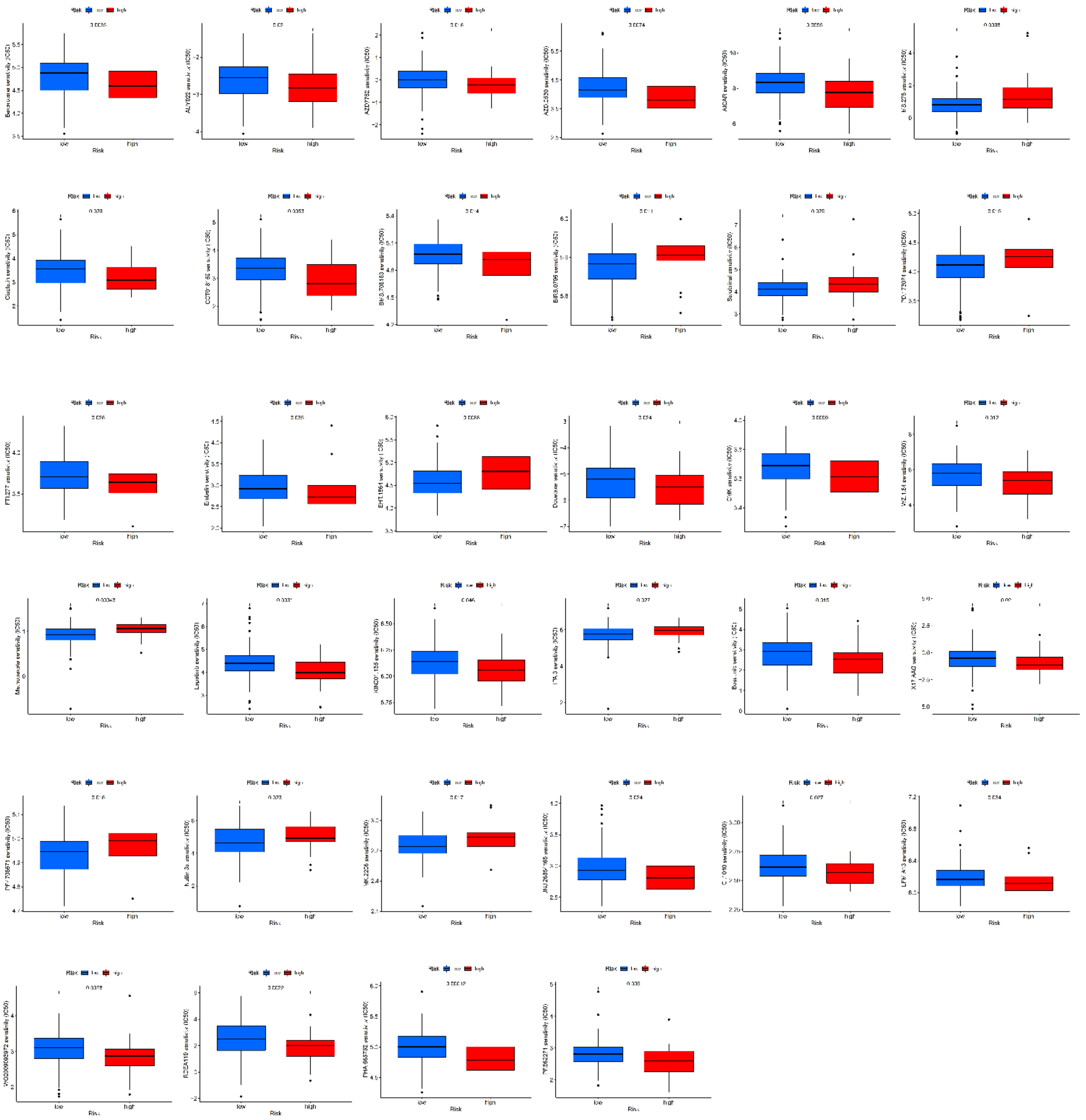

Supplementary Figure 7: The IC50 values of 48 chemotherapy drugs were different between the high- and low-risk groups of the test set

Supplementary Figures

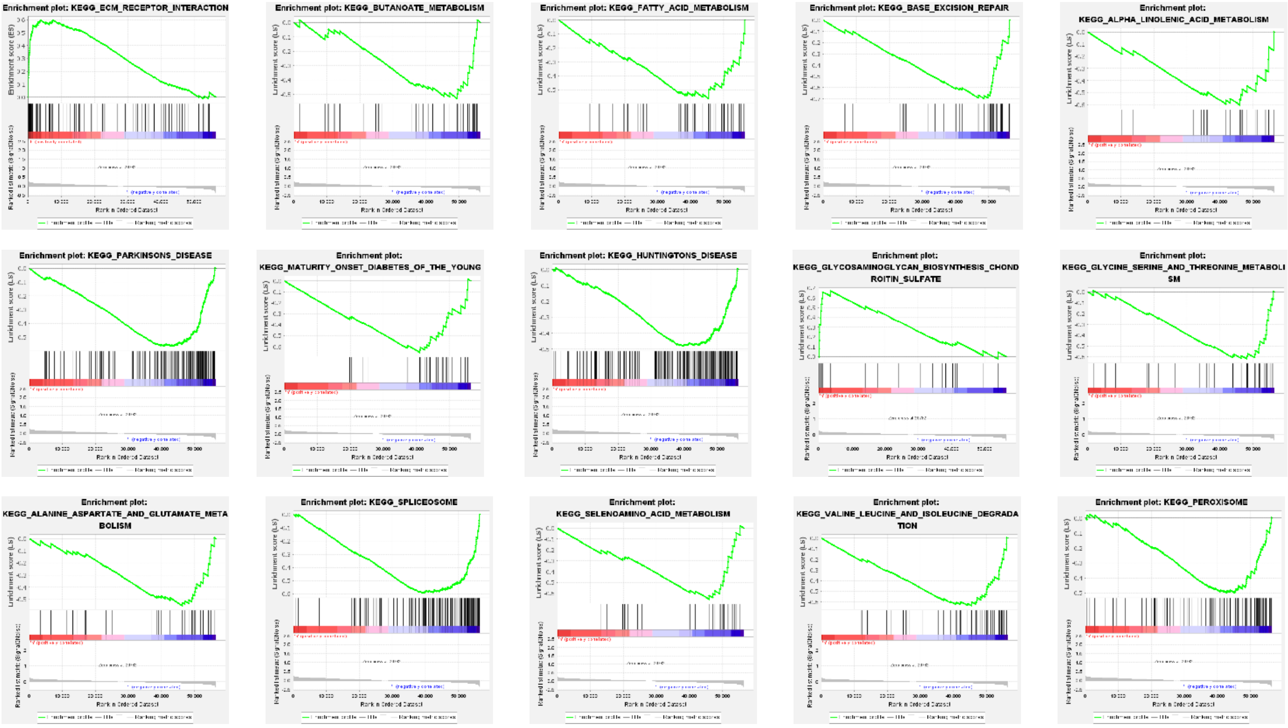

Supplementary Figure 8: GSEA of the training set

Supplementary Figures

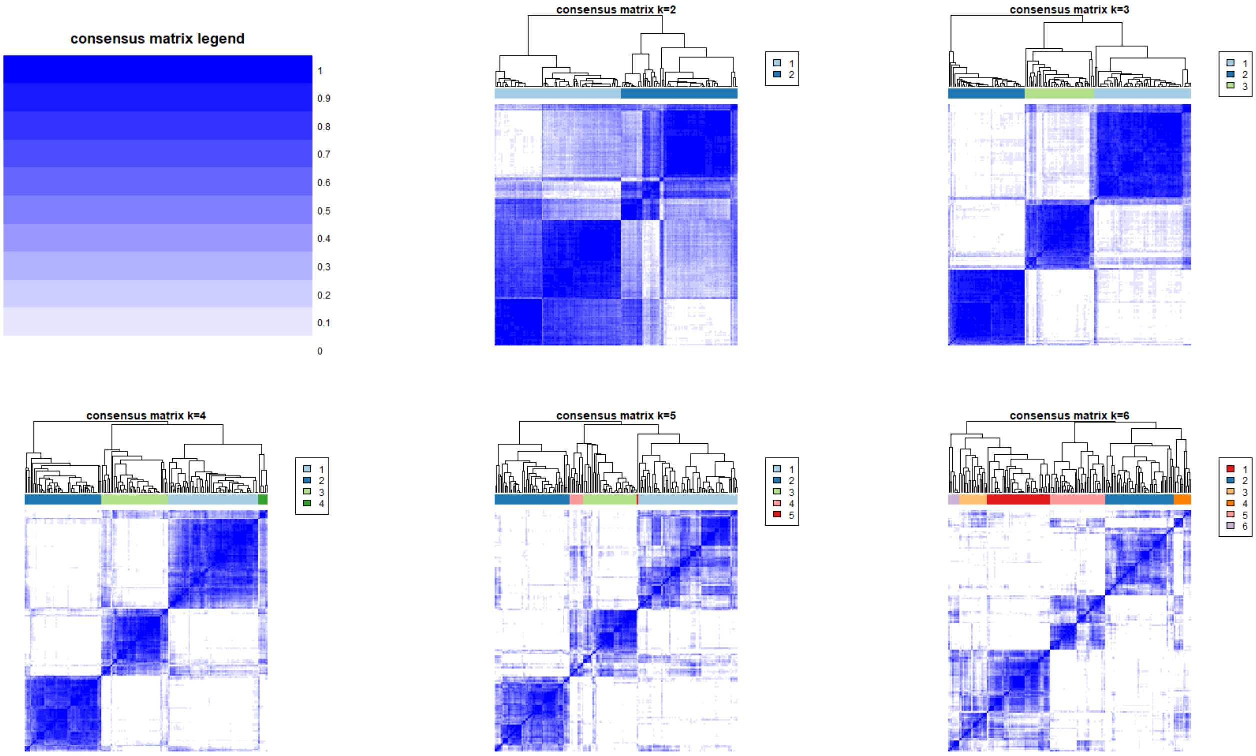

Supplementary Figure 9:Consensus matrixes of the training cohort for each k (k = 2–6), displaying the clustering stability using 1000 iterations of hierarchical clustering.

Supplementary Tables

Supplementary Table 1: The immune-related lncRNAs were identified by co-expression analysis (submitted as a separate Excel file).

Supplementary Tables

Supplementary Table 2: 53 pairs of DEIRlncRNAs were associated with prognosis

id	HR	HR.95L	HR.95H	pvalue
LINC01082|MIR100HG	0.533819	0.339581	0.83916	0.006533
AL161772.1|AC114488.1	1.875432	1.183742	2.971296	0.007397
AC009005.1|CALML3-AS1	0.505279	0.313828	0.813525	0.004965
AC009005.1|MIR100HG	0.534611	0.342673	0.834055	0.005787
LINC02474|AL158166.1	1.975968	1.178625	3.312715	0.009784
AC124319.1|AC091182.2	0.493781	0.312751	0.779597	0.002458
AC124319.1|AC114489.2	0.539881	0.344863	0.84518	0.007028
AC011503.2|AC129926.1	0.511303	0.321678	0.812709	0.004553
AC126178.1|G2E3-AS1	0.529103	0.340084	0.82318	0.00476
AC126178.1|AF127577.3	0.463024	0.294119	0.728928	0.000882
AC090229.1|AC073365.1	0.538514	0.341175	0.849995	0.007863
AL355916.1|MIR100HG	0.379244	0.206515	0.696445	0.001769
AL355916.1|AC073365.1	0.554972	0.354878	0.867886	0.00985
TDRKH-AS1|AC005180.2	0.534584	0.342819	0.833617	0.005731
TDRKH-AS1|AC112721.2	0.501152	0.316791	0.792805	0.003156
U62317.1|LINC01615	0.535696	0.339554	0.845138	0.007291
AC091182.2|AC067930.4	1.839671	1.174356	2.88191	0.007774
AC091182.2|LINC02163	1.883315	1.20213	2.950494	0.005715
AC091182.2|AL109615.3	2.122029	1.34973	3.336228	0.001118
AC010331.1|AC114489.2	0.448792	0.280784	0.717329	0.000813
AC010331.1|LINC01711	0.522289	0.325192	0.838846	0.007212
AC010331.1|AC129926.1	0.550637	0.3524	0.860389	0.008785
AC010331.1|AF127577.3	0.528224	0.334715	0.833607	0.006111
AC010331.1|AC073365.1	0.521403	0.323809	0.839574	0.007375
AC114489.2|AL109615.3	2.212552	1.408561	3.475453	0.000567
ZNF710-AS1|MIR100HG	0.509459	0.326722	0.794401	0.002926
AC114488.1|AC129926.1	0.452084	0.286862	0.712466	0.000624
AC114488.1|AF127577.3	0.392337	0.246836	0.623606	7.58E-05
AC114488.1|AC073365.1	0.396452	0.245401	0.640477	0.000157
AC105219.1|AC073365.1	0.506009	0.32133	0.796829	0.003279
AC092171.4|AF127577.3	0.515178	0.32704	0.811545	0.004228
AC092171.4|AC073365.1	0.516373	0.324239	0.822361	0.005374
LINC01614|AC025575.2	1.810037	1.160654	2.82275	0.008868
AC129926.1|SCAT2	1.930403	1.228557	3.033198	0.004334
AC129926.1|AATBC	2.151649	1.289884	3.589155	0.003335
AC129926.1|AL513218.1	1.80014	1.155916	2.803407	0.009294
AF127577.3|AC007938.3	2.107239	1.318064	3.368923	0.001849
AF127577.3|SCAT2	2.421608	1.508784	3.886696	0.000248
AF127577.3|AL513218.1	2.0158	1.283392	3.166179	0.002342
AC005180.2|AC010655.2	1.792111	1.150367	2.791858	0.009901
AC112721.1|AC067930.4	1.918493	1.199745	3.067832	0.006522
AC010655.2|LINC02544	0.538967	0.344264	0.843786	0.006879
AC007938.3|AATBC	1.828599	1.164982	2.870238	0.008695
C5orf66-AS1|AC073365.1	0.556981	0.357475	0.867831	0.009696
AC053503.3|LINC00460	1.939367	1.24154	3.02942	0.003606
AC026369.2|MIR100HG	0.503279	0.310845	0.814842	0.005225
SCAT2|CALML3-AS1	0.542767	0.344082	0.856179	0.008597
SCAT2|MIR100HG	0.533825	0.342576	0.831843	0.005546
MIR100HG|AATBC	1.871611	1.198365	2.923089	0.005861
AC012645.4|AC112721.2	0.519819	0.32781	0.824295	0.005413
AL109615.3|LINC02544	0.488991	0.311901	0.766627	0.001819
AL109615.3|AC007128.1	0.504294	0.318193	0.79924	0.003571
AATBC|LINC01605	0.484109	0.308958	0.758554	0.001546

Supplementary Table 3: 13 pairs of DEIRlncRNAs were used to construct the risk model

id	coef
`AC009005.1|CALML3-AS1`	-0.411528
`LINC02474|AL158166.1`	0.55009509
`AC124319.1|AC091182.2`	-0.4086088
`AC011503.2|AC129926.1`	-1.1341029
`AC126178.1|AF127577.3`	-0.5869197
`AL355916.1|MIR100HG`	-0.9119071
`AC091182.2|LINC02163`	0.69809231
`AC010331.1|AC114489.2`	-0.891699
`AC114488.1|AC073365.1`	-0.6302827
`AF127577.3|SCAT2`	0.60716484
`C5orf66-AS1|AC073365.1`	-0.6851894
`MIR100HG|AATBC`	0.46656428
`AL109615.3|AC007128.1`	-0.5002947

Supplementary Table 4: The detail comparison results of correlation ship between tumour infiltrating immune cells and risk score in the training set

immune	cor	pvalue
B cell_TIMER	-0.18811	0.011213
T cell CD8+_TIMER	0.276992	0.00016
Macrophage_TIMER	0.30891	2.33E-05
Myeloid dendritic cell_TIMER	0.159125	0.032384
B cell naive_CIBERSORT	0.15595	0.036047
T cell CD8+_CIBERSORT	-0.24814	0.000757
T cell follicular helper_CIBERSORT	-0.24736	0.000788
T cell regulatory (Tregs)_CIBERSORT	-0.16548	0.025993
NK cell activated_CIBERSORT	-0.17672	0.017321
Macrophage M0_CIBERSORT	0.178298	0.016332
Macrophage M2_CIBERSORT	0.232958	0.0016
B cell naive_CIBERSORT-ABS	0.164389	0.02701
Macrophage M0_CIBERSORT-ABS	0.184876	0.012719
Macrophage M2_CIBERSORT-ABS	0.26721	0.000276
Macrophage M1_QUANTISEQ	0.211048	0.004346
Macrophage M2_QUANTISEQ	0.1639	0.027475
T cell regulatory (Tregs)_QUANTISEQ	0.17144	0.02102
B cell_MCPCOUNTER	0.184183	0.013064
Monocyte_MCPCOUNTER	0.181334	0.014566
Macrophage/Monocyte_MCPCOUNTER	0.181334	0.014566
Myeloid dendritic cell_MCPCOUNTER	0.148585	0.045906
Endothelial cell_MCPCOUNTER	0.229063	0.001924
Cancer associated fibroblast_MCPCOUNTER	0.432067	1.25E-09
T cell CD4+ central memory_XCELL	-0.14891	0.045421
T cell CD8+ naive_XCELL	-0.30556	2.88E-05
T cell CD8+_XCELL	-0.19023	0.010318
Class-switched memory B cell_XCELL	-0.15237	0.040586
Endothelial cell_XCELL	0.187034	0.011697
Cancer associated fibroblast_XCELL	0.416902	5.28E-09
Hematopoietic stem cell_XCELL	0.31843	1.25E-05
Macrophage M1_XCELL	0.155796	0.036233
Monocyte_XCELL	0.183823	0.013246
T cell CD4+ Th1_XCELL	-0.23754	0.001283
stroma score_XCELL	0.360888	6.01E-07
microenvironment score_XCELL	0.22072	0.002828
Cancer associated fibroblast_EPIC	0.45026	2.02E-10
T cell CD4+_EPIC	-0.22766	0.002055
Endothelial cell_EPIC	0.196107	0.008149
NK cell_EPIC	0.172584	0.020166
uncharacterized cell_EPIC	-0.32945	5.94E-06

Supplementary Table 5: The detail comparison results of correlation ship between tumour infiltrating immune cells and risk score in the entire set

immune	cor	pvalue
B cell_TIMER	-0.24381	2.77E-06
T cell CD4+_TIMER	0.130765	0.012897
T cell CD8+_TIMER	0.294565	1.17E-08
Neutrophil_TIMER	0.168508	0.001311
Macrophage_TIMER	0.301734	4.92E-09
Myeloid dendritic cell_TIMER	0.18267	0.000487
B cell naive_CIBERSORT	0.105605	0.044948
T cell CD8+_CIBERSORT	-0.20837	6.63E-05
T cell follicular helper_CIBERSORT	-0.23731	5.15E-06
T cell regulatory (Tregs)_CIBERSORT	-0.11842	0.024441
Macrophage M0_CIBERSORT	0.160255	0.002258
Macrophage M1_CIBERSORT	0.131172	0.012617
Macrophage M2_CIBERSORT	0.278475	7.47E-08
Myeloid dendritic cell activated_CIBERSORT	-0.13222	0.011921
Neutrophil_CIBERSORT	0.167119	0.001439
B cell naive_CIBERSORT-ABS	0.143287	0.006389
T cell CD4+ memory resting_CIBERSORT-ABS	0.145706	0.005543
Macrophage M0_CIBERSORT-ABS	0.192504	0.000234
Macrophage M1_CIBERSORT-ABS	0.164333	0.001731
Macrophage M2_CIBERSORT-ABS	0.314404	1.01E-09
Mast cell activated_CIBERSORT-ABS	0.113464	0.031137
Neutrophil_CIBERSORT-ABS	0.181133	0.000544
Macrophage M1_QUANTISEQ	0.187296	0.000346
Macrophage M2_QUANTISEQ	0.175065	0.000836
T cell regulatory (Tregs)_QUANTISEQ	0.162123	0.002001
Myeloid dendritic cell_QUANTISEQ	-0.15348	0.003463
B cell_MCPCOUNTER	0.154482	0.003254
Monocyte_MCPCOUNTER	0.252538	1.17E-06
Macrophage/Monocyte_MCPCOUNTER	0.252538	1.17E-06
Myeloid dendritic cell_MCPCOUNTER	0.219531	2.58E-05
Endothelial cell_MCPCOUNTER	0.210466	5.57E-05
Cancer associated fibroblast_MCPCOUNTER	0.412306	3.01E-16
Myeloid dendritic cell activated_XCELL	0.189411	0.000296
T cell CD4+ central memory_XCELL	-0.12412	0.018316
T cell CD8+ naive_XCELL	-0.28263	4.68E-08
T cell CD8+_XCELL	-0.12821	0.014783
T cell CD8+ effector memory_XCELL	-0.10901	0.038439
Common myeloid progenitor_XCELL	0.134377	0.010591
Myeloid dendritic cell_XCELL	0.135861	0.009755
Endothelial cell_XCELL	0.162696	0.001927
Cancer associated fibroblast_XCELL	0.309524	1.87E-09
Granulocyte-monocyte progenitor_XCELL	0.176234	0.00077
Hematopoietic stem cell_XCELL	0.223985	1.74E-05
Macrophage_XCELL	0.199118	0.00014
Macrophage M1_XCELL	0.228937	1.12E-05
Monocyte_XCELL	0.275427	1.05E-07
T cell CD4+ Th1_XCELL	-0.1422	0.006807
T cell CD4+ Th2_XCELL	0.114779	0.029225
immune score_XCELL	0.151599	0.003888
stroma score_XCELL	0.279208	6.88E-08
microenvironment score_XCELL	0.236731	5.44E-06
Cancer associated fibroblast_EPIC	0.4031	1.55E-15
T cell CD4+_EPIC	-0.16973	0.001207
Endothelial cell_EPIC	0.181433	0.000532
Macrophage_EPIC	0.237533	5.04E-06
NK cell_EPIC	0.177453	0.000707
uncharacterized cell_EPIC	-0.30057	5.67E-09

Supplementary Table 6: The detail comparison results of correlation ship between tumour infiltrating immune cells and risk score in the test set

immune	cor	pvalue
B cell_TIMER	-0.30354	3.44E-05
T cell CD4+_TIMER	0.165164	0.026712
T cell CD8+_TIMER	0.317823	1.38E-05
Neutrophil_TIMER	0.281622	0.000128
Macrophage_TIMER	0.289468	8.10E-05
Myeloid dendritic cell_TIMER	0.212999	0.004094
T cell CD8+_CIBERSORT	-0.15344	0.039734
T cell follicular helper_CIBERSORT	-0.21321	0.004057
Macrophage M1_CIBERSORT	0.213163	0.004065
Macrophage M2_CIBERSORT	0.328915	6.54E-06
Myeloid dendritic cell activated_CIBERSORT	-0.20133	0.006726
Neutrophil_CIBERSORT	0.31496	1.66E-05
T cell CD4+ memory resting_CIBERSORT-ABS	0.202111	0.006511
NK cell activated_CIBERSORT-ABS	0.158755	0.033287
Macrophage M0_CIBERSORT-ABS	0.202358	0.006445
Macrophage M1_CIBERSORT-ABS	0.241243	0.001105
Macrophage M2_CIBERSORT-ABS	0.372912	2.53E-07
Neutrophil_CIBERSORT-ABS	0.33036	5.92E-06
Macrophage M1_QUANTISEQ	0.169906	0.022593
Macrophage M2_QUANTISEQ	0.191316	0.010091
NK cell_QUANTISEQ	-0.16405	0.027766
T cell regulatory (Tregs)_QUANTISEQ	0.148741	0.046287
Myeloid dendritic cell_QUANTISEQ	-0.25544	0.000539
cytotoxicity score_MCPCOUNTER	0.156313	0.036131
NK cell_MCPCOUNTER	0.190823	0.010289
Monocyte_MCPCOUNTER	0.332139	5.24E-06
Macrophage/Monocyte_MCPCOUNTER	0.332139	5.24E-06
Myeloid dendritic cell_MCPCOUNTER	0.299129	4.52E-05
Endothelial cell_MCPCOUNTER	0.202693	0.006356
Cancer associated fibroblast_MCPCOUNTER	0.395546	3.89E-08
Myeloid dendritic cell activated_XCELL	0.254716	0.000559
T cell CD8+ naive_XCELL	-0.25185	0.000649
Common myeloid progenitor_XCELL	0.153702	0.039396
Myeloid dendritic cell_XCELL	0.214164	0.003891
Endothelial cell_XCELL	0.154831	0.037955
Cancer associated fibroblast_XCELL	0.215721	0.003633
Granulocyte-monocyte progenitor_XCELL	0.202629	0.006373
Macrophage_XCELL	0.290754	7.51E-05
Macrophage M1_XCELL	0.315035	1.65E-05
Monocyte_XCELL	0.375999	1.98E-07
Plasmacytoid dendritic cell_XCELL	0.146857	0.049155
immune score_XCELL	0.224291	0.002471
stroma score_XCELL	0.210604	0.004543
microenvironment score_XCELL	0.257334	0.000488
Cancer associated fibroblast_EPIC	0.356946	8.72E-07
Endothelial cell_EPIC	0.171831	0.021084
Macrophage_EPIC	0.348024	1.69E-06
NK cell_EPIC	0.206503	0.005415
uncharacterized cell_EPIC	-0.28678	9.50E-05

Supplementary Table 7: IRlncRNAs were used to construct the risk model

id	coef	HR	HR.95L	HR.95H	pvalue
AC011468.1	-0.27946	0.75619	0.562733	1.016156	0.063788
AL031775.1	-0.2789	0.756612	0.519654	1.101622	0.145657
AL662844.4	-0.98401	0.37381	0.19978	0.699439	0.002082
AC005840.4	0.397718	1.488424	1.003746	2.207137	0.047865
AJ271736.1	-0.28866	0.74927	0.566442	0.99111	0.043121
NR2F1-AS1	0.629914	1.87745	1.303931	2.703224	0.000707
AL450384.2	-0.19587	0.822121	0.632655	1.068329	0.1428
PSMB8-AS1	-0.33045	0.718599	0.559672	0.922657	0.009565
AL354919.2	-0.26195	0.769548	0.617909	0.9584	0.019313
MAFG-DT	0.308731	1.361696	1.123587	1.650266	0.001643
ETV7-AS1	-0.35461	0.701444	0.433923	1.133895	0.147852
